# Agreement between Electrical Cardiometry and Pulmonary Artery Thermodilution for Measuring Cardiac Output in Isoflurane-Anesthetized Dogs

**DOI:** 10.3390/ani13081420

**Published:** 2023-04-21

**Authors:** Vaidehi V. Paranjape, Natalia Henao-Guerrero, Giulio Menciotti, Siddharth Saksena, Manuela Agostinho

**Affiliations:** 1 Department of Small Animal Clinical Sciences, Virginia-Maryland College of Veterinary Medicine, 205 Duck Pond Dr, Blacksburg, VA 24061, USA; nguerrer@vt.edu (N.H.-G.); giuliom@vt.edu (G.M.); manuagostinho@vt.edu (M.A.); 2 Department of Civil and Environmental Engineering, Virginia Polytechnic Institute and State University, 750 Drillfield Dr, Blacksburg, VA 24061, USA; siddha1@vt.edu

**Keywords:** canine, anesthesia, hemodynamics, monitoring, hemorrhage, hypovolemia, colloids, electrical velocimetry, noninvasive, blood transfusion

## Abstract

**Simple Summary:**

Using general anesthesia on animals causes significant changes to the heart and blood vessels that disrupt normal cardiovascular performance. Blood pressure (BP) is used for closely monitoring hemodynamics in anesthetized animals. However, BP is affected by various factors and may not always accurately correlate with the total body blood flow. Pulmonary artery thermodilution (PATD) is the gold standard for cardiac output (CO) measurements, but, due to the high risks associated with its invasiveness, it is not performed in clinical settings. This study evaluates noninvasive electrical cardiometry’s (EC) performance in measuring CO and other hemodynamic variables in healthy anesthetized dogs during acute blood volume manipulation. The EC measurements consistently underpredict the CO values as compared with PATD, but have a better performance when acute blood loss occurs. Even though the EC readings have a slightly higher error than the accepted error, this method is very good at showing trends in the CO measured using PATD. Other EC-derived variables are able to closely track the changes in the CO measured using PATD. In clinics, noninvasive EC may benefit the patient care quality for anesthetized dogs by monitoring the trends in hemodynamics and guiding anesthetists to diagnose and treat cardiovascular complications.

**Abstract:**

In animals, invasive pulmonary artery thermodilution (PATD) is a gold standard for cardiac output (CO) monitoring, but it is impractical in clinical settings. This study evaluates the agreement between PATD and noninvasive electrical cardiometry (EC) for measuring CO and analyzes the other EC-derived hemodynamic variables in six healthy anesthetized dogs subjected to four different hemodynamic events in a sequential order: (1) euvolemia (baseline); (2) hemorrhage (33% blood volume loss); (3) autologous blood transfusion; and (4) 20 mL/kg colloid bolus. The CO measurements obtained using PATD and EC are compared using Bland–Altman analysis, Lin’s concordance correlation (LCC), and polar plot analysis. Values of *p* < 0.05 are considered significant. The EC measurements consistently underpredict the CO values as compared with PATD, and the LCC is 0.65. The EC’s performance is better during hemorrhage, thus indicating its capability in detecting absolute hypovolemia in clinical settings. Even though the percentage error exhibited by EC is 49.4%, which is higher than the standard (<30%), EC displays a good trending ability. Additionally, the EC-derived variables display a significant correlation with the CO measured using PATD. Noninvasive EC may have a potential in monitoring trends in hemodynamics in clinical settings.

## 1. Introduction

General anesthesia is routinely required to perform a variety of surgical, medical, and diagnostic procedures in veterinary medicine. Anesthetic drugs can contribute to significant intraoperative hemodynamic alterations that can lead to further disturbances in cardiac performance [1,2]. In anesthetized dogs and cats, mean arterial blood pressure (MAP) < 60–65 mmHg is indicative of hypotension [3]. Anesthetists use blood pressure (BP) as a mere surrogate for circulatory function and often assume that targeting MAP > 60–65 mmHg prevents compromised perfusion and oxygen delivery to the organs. However, MAP is a product of cardiac output (CO) and systemic vascular resistance, suggesting that it is significantly impacted by both parameters. The literature available on animals highlights that a poor relationship can exist between CO and arterial BP [4,5]. Hence, if aggressive treatment for hypotension is initiated by entirely focusing on MAP values, detrimental effects on the cardiac workload and CO can occur, thus jeopardizing the quality of patient care. To accurately obtain a patient’s hemodynamic status, measuring the CO is critical; this is the variable characterizing the amount of blood ejected by the cardiac chambers in one minute [6].

Right heart catheterization is an invasive technique for evaluating the CO via pulmonary artery thermodilution (PATD), and has been considered the ‘gold standard’ since the 1970s [7]. In human medicine, its use has improved survival in high-risk surgical and critically ill patients by providing vital information regarding the CO, pulmonary artery (PA) pressure, PA occlusion pressure, and mixed venous saturation [8,9]. This method involves injecting a predetermined volume of cold saline into the PA catheter (Swan Ganz). The thermal variations resulting from the dilution are then measured. Using the blood temperature curve over time and the Stewart–Hamilton equation, the CO is calculated [7,8,9]. Despite the reported benefits of PATD, its use can lead to potential, although infrequent, complications that result from the catheter placement. Examples of these include arrythmias, infection, right ventricular perforation leading to cardiac tamponade, knotting or entanglement, PA rupture, infarction, thrombosis, and valvular trauma [10]. Hence, PATD’s utility in clinical settings seems impractical due to its invasiveness, risks, required skills, and equipment costs. Thus, there is demand for developing newer CO techniques that: (1) are minimally invasive or noninvasive; (2) provide continuous and reproducible measurements with a good level of agreement with the gold standard; (3) are user friendly with a fast response time; (4) are cost effective; and (5) are reliable during physiologic stress. Minimally invasive methods like lithium dilution, pulse contour analysis, pulse pressure analysis (e.g., pressure recording analytical method), and transesophageal echocardiography, and noninvasive methods like bioimpedance, bioreactance, transthoracic echocardiography, and modified Fick’s have been evaluated in veterinary medicine [11,12].

Electrical cardiometry (EC) is one of the newer noninvasive technologies for continuously measuring the CO and other EC-derived variables. With four skin electrodes, EC estimates the CO using the ‘electrical velocimetry^TM^’ algorithm, which assesses the cyclic variations in the thoracic electrical impedance during a cardiac cycle. The acceptable performance and accuracy of EC for conducting advanced hemodynamic monitoring has been reported in critically ill adult human patients [13], women receiving spinal anesthesia for cesarean delivery [14], children undergoing hemodialysis [15], and critical pediatric patients [16,17,18]. Even though some studies highlight EC’s high variability and its lack of agreement with transthoracic echocardiography [19], transpulmonary thermodilution [20], and PATD [21], it has shown promising results for closely tracking trends in COs [22,23]. There are limited studies on veterinary species that demonstrate EC’s utility in anesthetized dogs [24,25,26,27] and pigs [28]. Studies evaluating EC’s validity in dogs subjected to acute changes in COs are lacking. Hence, the specific aims of our study are: (1) evaluating the agreement of EC with the ‘gold standard’ PATD in measuring CO during acute blood volume manipulations; and (2) investigating the relationship between CO measured using PATD and EC-derived variables such as heart rate (HR_EC_), stroke volume (SV_EC_), thoracic fluid content (TFC), corrected flow time (FTC), stroke volume variation (SVV), contractility index (ICON™), variation in contractility (VIC™), systolic time ratio (STR), pre-ejection period (PEP), and left ventricular ejection time (LVET) during the induction and treatment of hypovolemia in isoflurane-anesthetized dogs. Our hypotheses are that: (1) the EC will display an acceptable agreement with PATD in this experimental scenario; and (2) the EC-derived variables will correlate with the variations in CO as measured using PATD in this canine hemorrhagic shock model.

## 2. Materials and Methods

### 2.1. Study Animals

This prospective, crossover, non-randomized experimental study utilized six adult purpose-bred, male, healthy Beagles (age 11–15 months; body weight 9.2 ± 0.5 kg) that were owned by the Virginia-Maryland College of Veterinary Medicine. The dogs were certified as ‘healthy’ by performing a thorough physical exam, complete blood count, and serum chemistry panel. They had free access to water, food, and enrichment toys, and were housed in a temperature- and light-cycle-controlled facility. The study design and procedures were approved by the Virginia Tech University-Institutional Animal Care and Use Committee (protocol number 20-235). Three weeks after the end of the study, all the dogs were successfully adopted into single family homes.

### 2.2. Anesthetic Induction and Standard Monitoring

The dogs were allowed 2 weeks to acclimatize to the laboratory environment. About 12 h prior to the experiment, food was withheld, but ad libitum access to the water was provided. On the day of the experiment, a 20-gauge, 2.95 cm catheter (BD Insyte Autoguard Shielded; Becton, Dickinson and Company, Franklin Lakes, NJ, USA) was aseptically inserted in the right cephalic vein, and preoxygenation was performed for 5 min using a fitted facemask with 4 L/min oxygen flow. Intravenous (IV) propofol (Propoflo 28; Zoetis Inc., Kalamazoo, MI, USA) was titrated to effect until endotracheal intubation was achieved. The endotracheal tube (7.5–8.5 mm ID Shiley^TM^ Cuffed Basic; Medtronic Animal Health, Minneapolis, MN, USA) was secured using a tie and connected to a ventilator-integrated anesthesia workstation (Datex-Ohmeda Aestiva 5/7900; GE Healthcare, Chicago, IL, USA) via a rebreathing system. Isoflurane (Fluriso; VetOne, Boise, ID, USA) in oxygen (1–2 L/min) was used for anesthetic maintenance with a targeted end-tidal concentration of isoflurane (ET_ISO_) of 1.6–1.8%. An infrared gas analyzer linked to a multiparameter monitor (Datex-Ohmeda S/5 Compact anesthesia monitor; GE Healthcare) was in place to continuously measure the ET_ISO_. The lead II electrocardiogram (ECG), heart rate (HR), esophageal temperature, partial pressure of end-tidal carbon dioxide (PETCO_2_), and peripheral oxygen saturation were also recorded using the same monitor. The body temperature was maintained between 36.7 and 38.1 °C throughout the experiment using a forced-air warming device (Bair Hugger; 3M Medical, St. Paul, MN, USA) and a circulating water blanket (TP700 T/Pump Warming and Cooling Therapy System; Gaymar Medical, Orchard Park, NY, USA). During the entire study period, no fluids were administered to eliminate the possibility of the fluid volume affecting the blood volume and skewing the hemodynamic data.

A 22-gauge, 2.54 cm catheter (BD Insyte Autoguard Shielded; Becton, Dickinson and Company) was used for catheterizing the dorsal pedal artery with the purpose of measuring the systolic, diastolic, and mean arterial blood pressures. The catheter was connected to a disposable pressure transducer (Deltran II; Utah Medical Products Inc., Midvale, UT, USA) via a saline-filled noncompliant short tubing and a 3-way luer lock stopcock, and the entire system was flushed with heparinized saline (3 units/mL). Prior to anesthetizing each dog, the transducer and the multiparametric monitor were calibrated using a digital pressure manometer. To assess the accuracy and linearity, the transducer was verified against the mercury manometer by use of a 2-point calibration technique (0 and 150 mmHg). The calibration was deemed acceptable when the difference between pressure exerted by the manometer and the displayed pressure on the multiparametric monitor was <2 mmHg. The transducer was situated at a height that approximately corresponded to the right atrium and zeroed to atmospheric pressure. Rocuronium (Rocuronium Bromide; Pfizer, New York City, NY, USA) was administered as an IV bolus 0.4 mg/kg followed by a constant rate infusion of 0.4 mg/kg/h to cause and maintain neuromuscular paralysis. The train-of-four supramaximal stimulation (Stimpod 450X; Xavant Technology, Pretoria, SA, USA) of the common peroneal nerve tested the blockade’s effectiveness. The dogs were mechanically ventilated using a volume-controlled ventilation mode with the tidal volume fixed at 12 mL/kg and the respiratory rate at 10–20 breaths/min to maintain PETCO_2_ between 30 and 40 mmHg. The dogs were then transitioned into the right lateral recumbency for EC instrumentation.

### 2.3. Instrumentation for EC to Measure CO (CO_EC_) and Other Hemodynamic Variables

**Figure 1:**
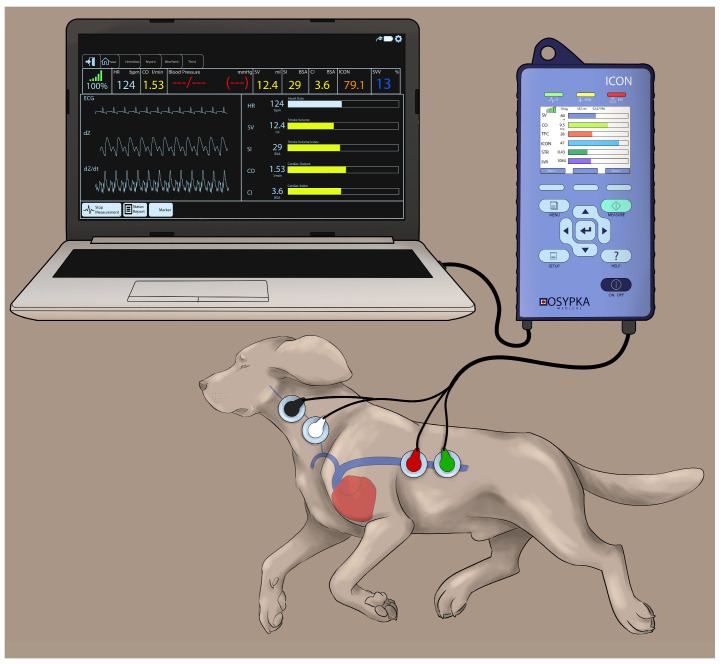
Cardiotronic electrode placement in a Beagle dog placed in the right lateral recumbency while using the electrical cardiometry (EC) monitor (ICON; Osypka Medical Inc., La Jolla, CA, USA). The electrodes are attached to an adhesive patch. The area on the left side of the neck adjacent to the common carotid artery and the left lower aspect of the thorax are clipped, thoroughly cleaned and dried before the application of the adhesive patch. The electrodes are connected to the ICON EC monitor by a cable and the monitor is synced with the laptop using an external communication cable to provide easy data management.

Electrical cardiometry using the ICON monitor (Osypka Medical Inc., La Jolla, CA, USA) provides continuous recordings of: (1) blood flow variables (i.e., CO_EC_, SV_EC_, and HR_EC_); (2) fluid status variables (i.e., TFC, SVV, and FTC); and (3) contractility variables (i.e., ICON™, VIC™, PEP, LVET, and STR). The area on the left aspect of the neck alongside the common carotid artery and the left lower aspect of the thorax were clipped and thoroughly cleaned to remove any dirt and skin debris. After ensuring the areas were dry, 4 Cardiotronic (Osypka Medical Inc.) electrocardiographic electrodes attached to adhesive patches were placed on the prepped skin areas, with 2 electrodes on the left aspect of the neck (at the level of common carotid artery) and 2 electrodes on the left thoracic area (at the level of the T8-T13 vertebrae corresponding to the location of the descending thoracic aorta) [24]. The electrodes were connected to the ICON EC monitor by a cable. The ICON EC monitor was then connected to the laptop with an external communication cable and synced with the laptop using the iControl™ software application (Osypka Medical Inc.) to provide easy data management (Figure 1).

**Figure 2:**
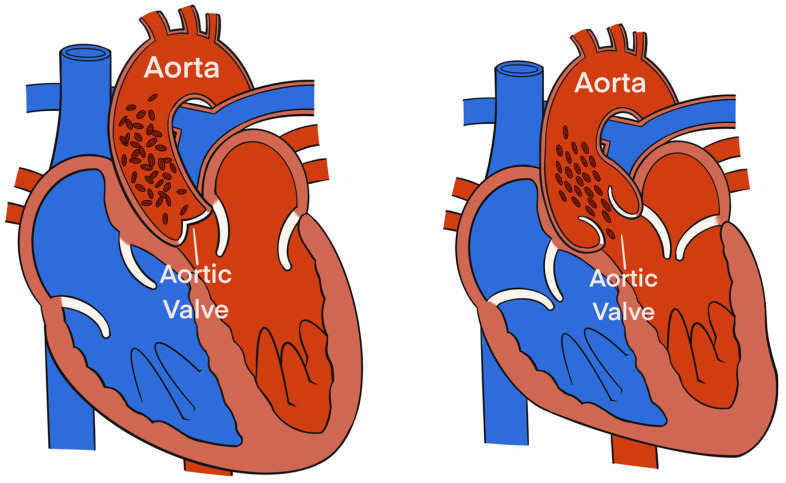
The alignment of the erythrocytes inside the aorta during a cardiac cycle induces significant variations in the impedance. During diastole, prior to aortic valve opening (**left**), the erythrocytes in the aorta are randomly oriented (due to no flow inside the aorta), which causes the applied electrical current to follow the circumference of the erythrocytes during their passage through the aorta resulting in a higher voltage and impedance measurement. During systole, after aortic valve opening (**right**), pulsatile flow causes the erythrocytes to parallelly align in the direction of the blood flow and the electrical current resulting in a lower impedance.

Electrical Velocimetry™ (Osypka Medical Inc.) is the physiological modeling and equation present in the ICON monitor that assesses the changes in the thoracic electrical bioimpedance during the cardiac systole and subsequent volumetric changes in the aorta that occur [28,29,30]. An electrical alternating current of constant amplitude is applied via the 2 outer electrodes to the thorax. The resultant voltage and surface ECG are provided by the 2 inner electrodes located at the neck. The ratio of the sensed voltage and applied current equals the thoracic electric bioimpedance that is recorded over time [28,29,30]. The Electrical Velocimetry™ model assumes that the erythrocytes’ alignment inside the aorta during a cardiac cycle causes significant variations in the impedance. During the diastole (prior to the aortic valve opening), the erythrocytes in the aorta are randomly oriented (no flow inside the aorta), which causes the applied electrical current to follow the circumference of the erythrocytes during their passage through the aorta, resulting in a higher voltage and impedance measurement. During the systole (after the aortic valve opening), the pulsatile flow causes the erythrocytes to parallelly align in the direction of the blood flow and the electrical current, resulting in a lower impedance (Figure 2) [28,29,30]. By analyzing the impedance’s rate of change before and after the aortic valve opening, the ICON algorithm derives SV_EC_ using the following equation:

$${\mathrm{SV}}_{\mathrm{EC}}=V_{\mathrm{EPT}}\times {\;V}_{\mathrm{FT}}\times \;\mathrm{LVET}$$

where V_EPT_ (mL) is the volume of electrically participating tissue calculated from the body mass and height indicating a patient constant, V_FT_ (s^−1^) is the ohmic equivalent of the mean aortic blood velocity during left ventricular ejection derived from the peak aortic blood flow acceleration, and LVET (s) is the left ventricular ejection time [28,29,30].

The CO_EC_ (L/min) is then calculated as the product of SV_EC_ and HR recorded using EC (HR_EC_). The physiologic model in the ICON monitor then correlates the measured changes in thoracic electrical bioimpedance to derive other hemodynamic variables such as TFC, FTC, ICON™, VIC™, STR, PEP, and LVET. The CO_EC_, SV_EC_, and HR_EC_ are averaged over a 1 min interval as set on the internal database. To ensure the EC data’s reliability, the HR_EC_ values are verified against the pulse rate from the pulse–oximetry and arterial pressure waveforms as well as the HR from the ECG before every recording of the CO_EC_, SV_EC_, HR_EC_, and other EC-derived hemodynamic variables. Moreover, to maintain the data accuracy, the EC-derived values are only recorded when the signal quality index displayed on the ICON monitor is 100. The SVV calculation is automatically performed using the ICON internal software with the following formula and is simultaneously noted at each datapoint as an average during 1 min:

$$\mathrm{SVV}\;\left(\%\right)=\frac{{\mathrm{SV}}_{\max}-{\mathrm{SV}}_{\min}}{({\mathrm{SV}}_{\max\;}+{\mathrm{SV}}_{\min\;})/2}\times 100$$

where SV_max_ and SV_min_ are the maximum and minimum stroke volume (mL), respectively, over one respiratory cycle.

The PEP (ΔPEP) variation is manually calculated by noting the maximal and minimal PEP values during inspiration and expiration, respectively, during one respiratory cycle. These measurements are repeated over three different respiratory cycles (yielding three values at inspiration and three values at expiration) and are each averaged. The formula used for the ΔPEP calculation is as follows [31,32]:

$$\Delta \mathrm{PEP}\;\left(\%\right)=\frac{{\mathrm{PEP}}_{\max}-{\mathrm{PEP}}_{\min}}{({\mathrm{PEP}}_{\max\;}+{\mathrm{PEP}}_{\min\;})/2}\times 100$$

where PEP_max_ and PEP_min_ are maximum and minimum pre-ejection periods (ms), respectively, over one respiratory cycle.

### 2.4. Instrumentation for CO Monitoring by PATD (CO_PATD_)

The dogs were then transitioned to dorsal recumbency for PATD instrumentation and remained in this position for the entire study period. Due to this change in the body position, the arterial pressure transducer was re-zeroed to atmospheric pressure. Using the modified Seldinger technique, a 5 Fr 13 cm double lumen central venous catheter (MILA International Inc., Florence, KY, USA) was aseptically placed in the left jugular vein. This catheter’s function was to draw a fixed blood volume to induce acute hemorrhagic shock, and to transfuse blood and Hydroxyethyl Starch during hemorrhage treatment. The same technique was used to place a 6 Fr 8.5 cm hemostasis introducer (Fast-Cath; Abbott Cardiovascular, Plymouth, MN, USA) in the right jugular vein, through which a 5 Fr 75 cm PA thermodilution Swan Ganz catheter (132FS; Edwards Lifesciences Corp., Irvine, CA, USA) was advanced by pressure–waveform guidance until its distal tip was located in the PA. The accurate positioning of the distal and proximal ports was confirmed by observing the characteristic pressure waveforms and pressure values of the main pulmonary artery and right atrium, respectively, upon connection with the CO monitor (Carescape B850; GE Healthcare, Chicago, IL, USA). The proximal injectate port was used for injecting the cold saline bolus during the CO_PATD_ determination. The catheter’s proximal and distal ports were connected to another set of disposable pressure transducers (Deltran II, Utah Medical Products Inc.), and these were calibrated and positioned in a similar manner to the arterial pressure transducer. The appropriate computation constant was selected on the monitor screen based on the catheter model, volume, and injectate’s temperature as recommended by the manufacturers [33].

Each CO_PATD_ reading was timed at the end of the expiration, and a 3 mL bolus of chilled (from 2 °C to 5 °C) 0.9% sodium chloride solution was injected over <3 s into the proximal port of the Swan Ganz catheter. At each data timepoint, a CO reading corresponded to the mean of 3 consecutive measurements within 10% variation. The injections were always manually administered by the same person and spaced at least 90 s apart. Three researchers (V.V.P., N.H.G., and G.M.) were assigned to collect the specific data and were blinded to the readings obtained by the others.

### 2.5. Data Collection during Acute Change in Hemodynamics

**Figure 3:**
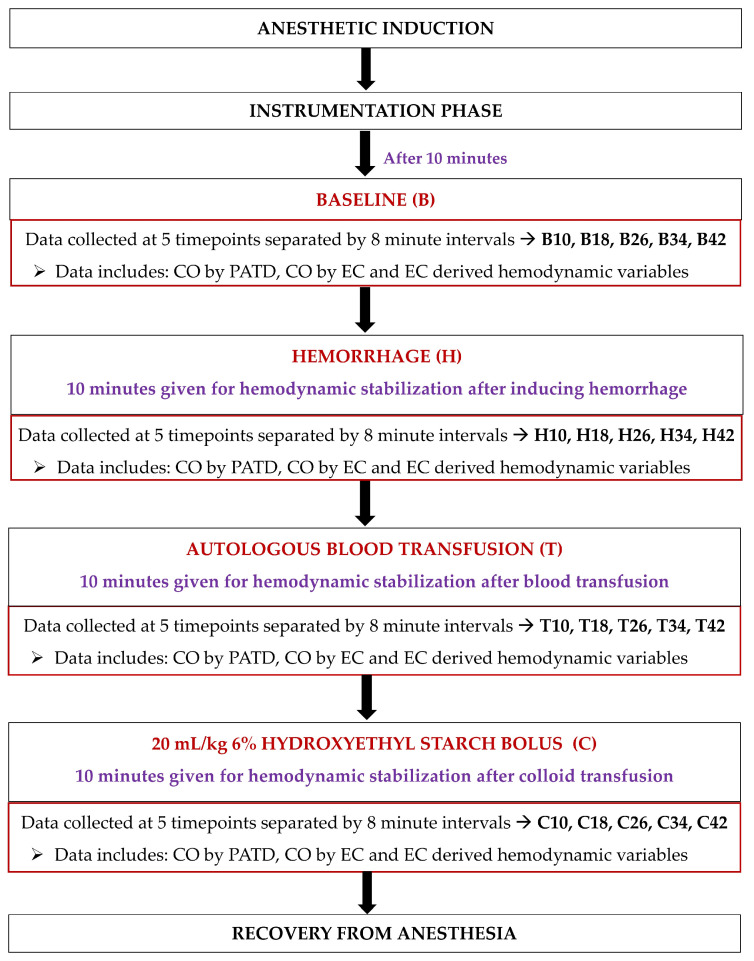
Timeline of the study design and data collection in six, healthy, adult isoflurane-anesthetized Beagle dogs. After anesthetic induction and instrumentation, data were collected: (1) at the baseline; (2) after 33% blood volume loss (H); (3) after the autologous blood transfusion (T); and (4) after the infusion of 20 mL/kg 6% Hydroxyethyl Starch 130/0.4 in 0.9% sodium chloride solution (C). A 10 min hemodynamic stabilization period was provided between the manipulation of blood volume during H, T, and C, and before data collection.

Upon completing instrumentation, each dog was subjected to a sequential non-randomized experimental design to induce acute changes in the blood volume (Figure 3). Ten minutes after the completion of the instrumentation, the baseline (B) data collection was performed at five timepoints separated by 8 min intervals (B10, B18, B26, B34, and B42). Once the readings were performed, hemorrhage (H) was initiated by withdrawing 33% of the estimated total circulating blood volume (considered as 90 mL/kg of body weight [34]) from the left jugular catheter over a period of 15 min. After 10 min of hemodynamic stabilization, the hypovolemic state data were collected at five timepoints separated by 8 min intervals (H10, H18, H26, H34, and H42). The blood volume drawn was then stored in blood collection bags coated with an anticoagulant (CPDA-1 Blood Collection System; Animal Blood Resources International, Stockbridge, MI, USA). The blood-filled bags were placed on a weighing scale and were simultaneously weighed for the exact amount of blood removed during hemorrhage. The next step involved an autologous transfusion of the total blood volume previously withdrawn via the left jugular catheter over a period of 15 min using an infusion pump (Alaris Carefusion; BD, Franklin Lakes, NJ, USA). Once 10 min elapsed, the data collection was performed at five timepoints separated by 8 min intervals (T10, T18, T26, T34, and T42). Lastly, a 20 mL/kg 6% Hydroxyethyl Starch (VetStarch 130/0.4 in 0.9% sodium chloride; Zoetis Inc.) bolus was administered in the left jugular vein over a period of 15 min via the same infusion pump and, after 10 min of stabilization, the data were acquired at five timepoints separated by 8 min intervals (C10, C18, C26, C34, and C42).

### 2.6. Anesthetic Recovery and Post Experiment Monitoring

When the final data were acquired, the rocuronium infusion was discontinued. The jugular and arterial catheters were removed, and recovery was initiated by turning off the isoflurane vaporizer. Upon extubation, 0.3 mg/kg IV methadone was administered to all dogs and they were transferred to individual cages; their cardiopulmonary parameters and catheter sites were monitored hourly for the first 4 h, every 3 h for the next 8 h, and every 8 h for a total of 96 h. The pain scores were assessed using the Glasgow composite pain scale short form [35] and evaluation of the HR, respiratory rate and depth, and non-invasive blood pressure measurements was performed. Additional 0.3 mg/kg IV methadone was used as a rescue analgesic when needed.

### 2.7. Statistical Analysis

Using a literature search of veterinary studies comparing CO measurement techniques with the gold standard PATD [33,36,37], an a priori power analysis indicates that a sample size of six dogs is necessary to show a minimum 20% significant difference in CO during the induction of acute changes in hemodynamics carried out by the manipulation of blood volume, assuming a statistical power of 0.8 and an alpha level of 0.05 (http://estatistica.bauru.usp.br/calculoamostral/ accessed on 1 September 2021).

The Shapiro–Wilk and D’Agostino–Pearson tests were used to assess the physiological variables at each time point and the hemodynamic event. The data for all normally distributed variables were reported as mean ± standard deviation (SD). To compare the differences across the variables during the hemodynamic event occurrences, a one-way analysis of variance for repeated measures was performed.

A Tukey’s Honest Significant Difference (post hoc test) was applied to the paired comparisons and, if there was a lack of sphericity, Greenhouse and Geisser corrections were performed. This was followed by a pairwise *t* test (parametric data) or Wilcoxon Signed Rank test (non-parametric data) to compare the differences among the paired samples. The correlation between CO_PATD_ and several variables (e.g., SV_EC_, TFC, FTC, SVV, ΔPEP, ICON^TM^, VIC^TM^, STR, PEP, and LVET) was assessed using the least squares regression analysis. The statistical analysis was carried out using SAS Version 9.4 (SAS Institute Inc., Cary, NC, USA) and *p* < 0.05 values were considered statistically significant.

For calculating the bias for each observation, the CO_PATD_−CO_EC_ difference was used. Since the CO measurements being evaluated were distributed over a wide physiologic range and there was a lack of normality exhibited by some variables, the bias was calculated. The bias was expressed as a percentage of the average CO values, as previously reported [38]; the formula for the relative bias (RB) was as follows:

$$\mathrm{Relative}\;\mathrm{Bias}\;\left(\mathrm{RB}\right)=\frac{\left({\mathrm{CO}}_{\mathrm{PATD}}-{\;\mathrm{CO}}_{\mathrm{EC}}\right)}{\left[0.5\times \left({\mathrm{CO}}_{\mathrm{PATD}}+{\;\mathrm{CO}}_{\mathrm{EC}}\right)\right]}\times 100\;$$

Here, a positive RB (%) value suggests that the CO value was underpredicted by the EC when compared with the PATD, and a negative RB (%) indicates that the CO_EC_ was overpredicted compared to the CO_PATD_. The limits of agreement (LOA) were calculated as RB ± 1.96 × SD to include a 95% confidence interval (CI). As per the previously published standard for comparing the CO methods, the overall RB was required to be <30% for an acceptable performance using the test method (EC) when compared to the gold standard method (PATD). Additionally, the absolute value of RB for each observation was compared with the 30% value to establish an estimate for the overall performance.

A linear regression analysis was conducted to evaluate the correlation in the measurements between the CO_PATD_ and CO_EC_, and the Lin’s concordance correlation$\;(\rho_{c}$) was also evaluated between the PATD and EC to measure the test method’s data reproducibility [39]. Bland–Altman (BA) analysis was performed to demonstrate the agreement between the CO_PATD_ and CO_EC_ values [40]. When the mean and SD of the bias between the CO_EC_ and CO_PATD_ was influenced by the original measurement’s magnitude (proportioning effect), the BA analysis for non-uniform differences was performed. For such instances, the bias was linearly regressed against the average bias [41]. A polar plot analysis was used to illustrate the CO_EC_‘s trending ability along with investigating the agreement between the CO_PATD_ and CO_EC_ [42,43]. The distance from the center represents the absolute values of the mean change in ($\left[∆{\mathrm{CO}}_{\mathrm{PATD}}+∆{\mathrm{CO}}_{\mathrm{EC}}\right]/2$) and the angle with the horizontal (0° radial) indicates disagreement. A good trend is reflected by the data located within 10% of the mean CO values.

## 3. Results

### 3.1. Success of Data Collection and Monitoring of the Study Dogs during the Experiment

The anesthetic induction and maintenance were uneventful in all dogs and there was a smooth emergence from the general anesthesia during recovery. In each dog, successful placement of the Swan Ganz thermodilution catheter was performed without any complications. No missing data for the PATD and EC were reported, and all dogs successfully completed the experiment. The dogs were normothermic throughout the experiment (37.6 ± 0.9 °C) as well as during the recovery period. After completion of the study, the jugular and arterial catheter sites did not show any evidence of substantial hematoma or subcutaneous bruising. Two out of the six dogs required an additional dose of 0.3 mg/kg IV methadone because of the pain scores at the 10 h timepoint post-extubation. The cardiorespiratory parameters, appetite, demeanor, and excretory functions were within the normal limits throughout the 96 h post extubation period. There were no differences in the total anesthesia time (*p* = 0.88), time spent in hemorrhagic shock (*p* = 0.39), and time spent during blood transfusion (*p* = 0.22) and colloid administration (*p* = 0.26) between all the dogs.

### 3.2. Comparisons between CO_PATD_ and CO_EC_ during the Experiment

For each dog, a pair of CO measurements was obtained using the PATD and EC techniques for each of the five timepoints during baseline, hemorrhage, autologous blood transfusion, and colloid administration. Twenty pairs of CO measurements were collected for each dog and, overall, 120 pairs of measurements were obtained from all six dogs. The mean *±* SD for the CO_PATD_ and CO_EC_ during acute blood volume manipulations are shown in Table 1. Since the values for the CO_PATD_ and CO_EC_ for the five timepoints under each event are not statistically significant, the mean *±* SD values are calculated from the timepoint measurements to represent each event. The CO measurements using PATD and EC are significantly decreased during hemorrhage as compared with the baseline values (*p* = 0.036), but they significantly improved after the autologous blood transfusion (*p* = 0.041). After a colloid infusion, both methods detect a significant rise in the CO values (*p* = 0.032).

The mean ± SD of the bias between the two methods (CO_PATD_ − CO_EC_) is 0.55 ± 0.38 L/min. The mean ± SD of the relative bias (%) between the two methods is 27.7 ± 16.8% (LOA: from −5.1% to 60.5%). Approximately fifty percent (50%) of the 120 observations have an absolute value of relative bias >30%. The percentage error expressed as (1.96 × SD of the bias/mean CO) × 100 is calculated as 49.4%, which is not within the acceptable range as proposed in the literature [34]. To compare the EC’s performance across the different hemodynamic events, the mean ± SD (LOA) of the relative bias is reported as 33.6 ± 4.4% (from 24.9% to 42.4%) for baseline, 4.9 ± 12% (from −18.8 to 28.7%) for hemorrhage, 35.6 ± 7.3% (from 21.1 to 49.9%) for blood transfusion, and 36.7 ± 14.5% (from 8.1 to 65.2%) for colloid infusion. By analyzing the mean ± SD of the relative bias across all events, it is evident that the EC consistently underpredicts the PATD measurements, but EC’s performance is significantly better during hemorrhage.

**Figure 4:**
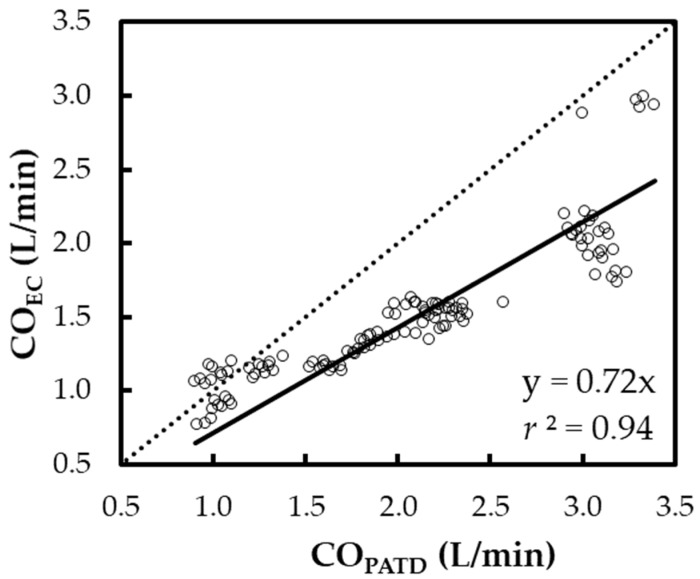
Scatter plot representing the cardiac output (CO) measurements using electrical cardiometry (CO_EC_) and pulmonary artery thermodilution (CO_PATD_) for six anesthetized Beagle dogs across five timepoints during four hemodynamic events (baseline, hemorrhage, autologous blood transfusion, and colloid infusion), thus yielding 120 paired observations (circles). Regression analysis about the line Y = X (dashed line) resulted in a good fit (solid line) as shown by the slope (0.72) and *r*^2^ (0.94).

The regression in line Y = X (Figure 4) yields the expression: CO_EC_ = 0.72 × CO_PATD_ (*r*^2^ = 0.94). A positive mean relative bias (27.7%) and slope (<1) about Y = X indicates the EC underpredicts the CO when compared with the PATD. The Lin concordance correlation coefficient between the CO_PATD_ and CO_EC_ is *ρ*_c_ = 0.65 (*p* < 0.001).

**Figure 5:**
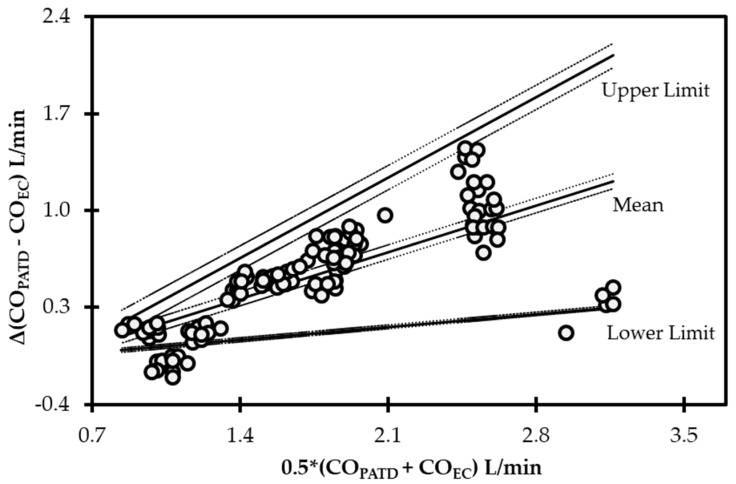
Bland−Altman analysis for non-uniform differences using cardiac output (CO) values measured using electrical cardiometry (CO_EC_) and pulmonary artery thermodilution (CO_PATD_) techniques in anesthetized Beagle dogs (*n* = 6) across five timepoints during four hemodynamic events (baseline, hemorrhage, autologous blood transfusion, and colloid infusion), thus yielding 120 paired observations (circles). Each circle represents an individual difference value corresponding to an average value and mean shows a strong positive bias (slope = 0.48; intercept = −0.31) indicating underprediction. As displayed, the solid lines indicate the mean and upper and lower limits of agreement, and the dashed lines indicate the 95% confidence intervals around these values.

**Figure 6:**
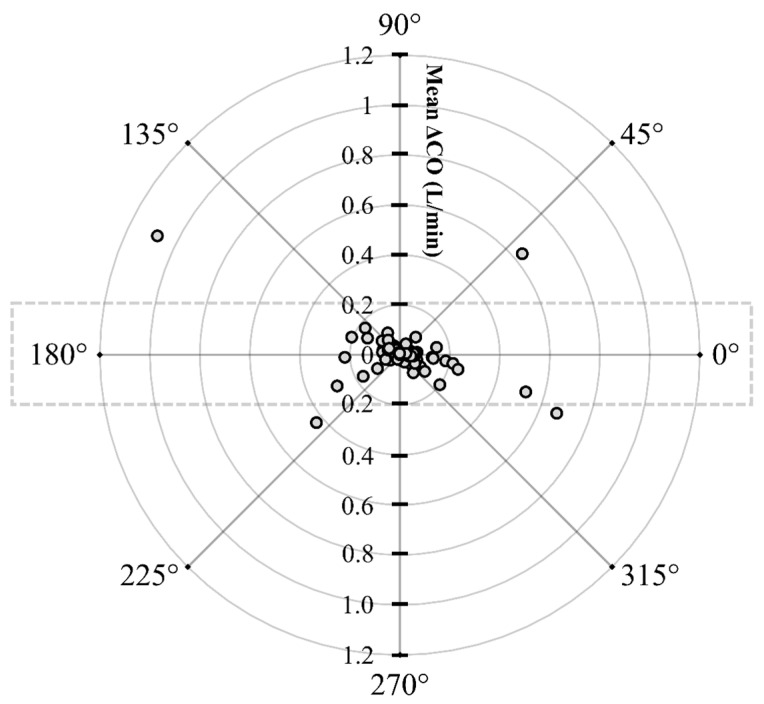
Polar plot displaying changes in cardiac output (CO) measured using electrical cardiometry and pulmonary artery thermodilution methods in anesthetized Beagle dogs (*n* = 6) across five timepoints during four hemodynamic events (baseline, hemorrhage, autologous blood transfusion, and colloid infusion), thus yielding 120 paired observations (circles). Dotted lines indicate 10% boundaries (i.e., 10% = 0.206 L/min as mean CO = 2.06 L/min). The distance from the center reflects the absolute values of the mean change in CO ($\left[∆{\mathrm{CO}}_{\mathrm{PATD}}+∆{\mathrm{CO}}_{\mathrm{EC}}\right]/2$), and the angle with the horizontal (0° radial axis) is indicative of a lack of agreement. The polar plot analysis exhibits good trending ability as only four points are located on the exterior of the limits of good agreement.

The initial data analysis reveals that the bias (CO_PATD_ − CO_EC_) is normally distributed as reported by the Shapiro–Wilk test and the D’Agostino–Pearson test. Considering there is a large variation in the mean relative bias across different hemodynamic events, a BA analysis for non-uniform differences was conducted. This analysis shows a positive trend (slope = 0.48; intercept = −0.31) between the bias and the average CO data (Figure 5) that are interpreted as a uniform underestimation of EC across the fluctuating CO values. The polar plot analysis reveals a good trending pattern across the wide range of CO values as most data points are located within the limits of good agreement (i.e., 10% = 0.206 L/min as mean CO = 2.06 L/min) and only four points are on the exterior of these limits (Figure 6). Although the BA analysis indicates a consistent positive bias (underestimation) using EC, this technique especially exhibits a good agreement during hemorrhage and an overall good trending ability.

### 3.3. Relationship between CO_PATD_ and EC-Derived Variables Denoting Blood Flow and Fluid Status

**Figure 7:**
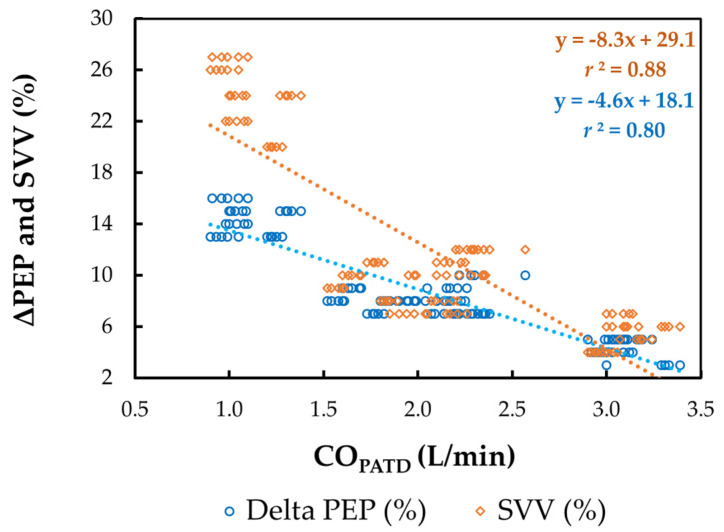
Scatterplot of cardiac output (CO) measured using pulmonary artery thermodilution (CO_PATD_) versus stroke volume variation (SVV) and variation in pre-ejection period (ΔPEP) in six healthy, isoflurane-anesthetized Beagle dogs across five timepoints during four hemodynamic events (baseline, hemorrhage, autologous blood transfusion, and colloid infusion), thus yielding 120 paired observations. Th orange dotted line with orange solid diamonds represents the best-fit correlation for SVV and the blue dotted line with blue solid circles represents the best-fit correlation for ΔPEP.

The mean *±* SD for HR_EC_, SV_EC_, TFC, FTC, SVV, and ΔPEP during four different hemodynamic events are shown in Table 2. A significant reduction in CO_PATD_ values during hemorrhage coincided with a significant increase in HR_EC_ (*p* = 0.038), SVV (*p* = 0.015), and ΔPEP (*p* = 0.024), with a simultaneous decrease in SV_EC_ (*p* = 0.021), TFC (*p* = 0.019) and FTC (*p* = 0.014). Following the administration of blood, as the CO_PATD_ stabilized, the HR_EC_, SVV, and ΔPEP significantly reduced (*p* < 0.001) and SV_EC_, TFC, and FTC were significantly higher compared to the values during hemorrhage. After the colloid infusion, as there was a further increment in the CO_PATD_ values, the HR_EC_, SV_EC_, TFC, and FTC show significant changes in the same direction as the CO_PATD_ (*p* < 0.001). In contrast, the SVV and ΔPEP values decline significantly (*p* < 0.001). The changes in the CO_PATD_ are significantly (*p* < 0.001) correlated with SV_EC_ (*r*^2^ = 0.91), TFC (*r*^2^ = 0.83), FTC (*r*^2^ = 0.89), SVV (*r*^2^ = 0.88; Figure 7), and ΔPEP (*r*^2^ = 0.80; Figure 7).

### 3.4. Relationship between CO_PATD_ and EC-Derived Variables Denoting Cardiac Contractility

The mean *±* SD for ICON^TM^, VIC^TM^, STR, PEP, and LVET during four different hemodynamic events are shown in Table 3. A significant fall in the CO_PATD_ during the blood volume depletion is associated with a significant decrease in ICON^TM^ (*p* = 0.023), VIC^TM^ (*p* = 0.029), and LVET (*p* = 0.010), while the STR (*p* = 0.015), and PEP (*p* = 0.018) are significantly higher. Once the dogs were administered the blood transfusion and the colloid infusion, ICON^TM^, VIC^TM^, and LVET significantly increased, and the STR and PEP were significantly reduced as compared with the values during hemorrhage (*p* < 0.001). The changes in the CO_PATD_ are significantly (*p* < 0.001) correlated with ICON^TM^ (*r*^2^ = 0.82), VIC^TM^ (*r*^2^ = 0.78), STR (*r*^2^ = 0.81), PEP (*r*^2^ = 0.84), and LVET (*r*^2^ = 0.87).

## 4. Discussion

Even though PATD is considered the de facto ‘gold standard’ in human and veterinary medicine [8,9,33,36,37], it is linked with well-known limitations [44] and complications [10]. The factors contributing to measurement errors and variability include injectate volume and temperature, intracardiac shunts, valvular dysfunction, catheter dead space, injection time, irregular respiratory patterns, concurrent IV infusions, and exogenous cooling or warming [44]. These limitations and adverse effects have led to advancing minimally or noninvasive CO measurement methods. The technique, when undergoing testing and validation, must demonstrate safety and offer reliable and repeatable readings during a wide range of clinically occurring CO scenarios, i.e., euvolemia, hypovolemia, and hypervolemia. This study’s results show a consistent EC underperformance across a broad range of CO values occurring in four different hemodynamic events. Approximately fifty percent (50%) of the 120 observations have an absolute value of relative bias >30%, with a concordance of 0.65 between the CO_PATD_ and CO_EC_. The overall 49.4% error is not within the acceptable range (<30%) published as a standard [38]. Interestingly, the agreement between PATD and EC was better during the hemorrhage, and the test method exhibits an overall good trending ability.

While performing a method comparison study on CO monitoring, using the right tools to fairly assess the technology is important. Some recommended steps [43,45,46] are: (1) using a reliable reference standard; (2) evaluating the test method over a wide range of values and conditions; and (3) performing data analysis using a combination of statistical approaches such as the calculation of bias, LOA and percentage error, generation of scatter plots, four-quadrant plots, and polar plot analysis. Additionally, the test method must also meet the criteria for acceptability of agreement, which is a percentage error of 30% or less [38], so that the results can be deemed comparable and applicable between studies and study populations across wide ranges of CO values. From a clinician’s standpoint, apart from accuracy, other elements such as the safety, convenience, adaptability, level of invasiveness, and cost of instrumentation should also be considered. In our study, even though the EC consistently underpredicts the CO_PATD_ values and demonstrates a lower concordance with PATD and a higher percentage error, it shows a very good trending ability throughout the wide range of CO values studied during the hemodynamic events. Moreover, it performs better when the dogs suffer from acute absolute hypovolemia. In clinical scenarios, clinicians may accept a CO method that may not provide highly accurate absolute measurements but is still able to reliably detect trends whenever CO values change [22,23]. They may also prefer less invasive techniques rather than dealing with PATD’s potential serious complications and consequent poor outcomes. Based on a meta-analysis, a percentage error in agreement with the PATD of ±45% signifies a more realistic approach of gaining precision in clinical settings [47]. Accounting for a potential time delay when comparing between two CO measurement technologies and trending analysis may be worthwhile. Even though PATD is a gold-standard method, its response time is longer than EC, which estimates CO values in real time, so this specific feature needs scrutiny.

Our study shows the mean ± SD of the bias between the two techniques (CO_PATD_ − CO_EC_) is 0.55 ± 0.38 L/min. The mean ± SD of the relative bias (%) between the two methods is 27.7 ± 16.8% (LOA: from −5.1% to 60.5%). When comparing EC’s performance across the different hemodynamic events, the mean ± SD (LOA) of the relative bias is reported as 33.6 ± 4.4% (from 24.9% to 42.4%) for the baseline, 4.9 ± 12% (from −18.8 to 28.7%) for the hemorrhage, 35.6 ± 7.3% (from 21.1 to 49.9%) for the blood transfusion, and 36.7 ± 14.5% (from 8.1 to 65.2%) for the colloid infusion. Although the EC consistently underpredicts the PATD measurements, its performance is significantly better during the acute hemorrhagic shock. We speculate that the significant increase in CO observed after blood transfusion and colloid infusion may have resulted in higher velocity of blood flow in vessels. This combined with low blood viscosity during the hyperdynamic state may have disrupted the laminar flow and converted it to turbulent flow by increasing the critical Reynolds number. This chaotic blood flow could have impacted the orientation of the erythrocytes and changes in bioimpedance, thus contributing to inaccuracies when higher CO values were measured. When the CO_EC_ values are compared with the CO values measured using transpulmonary thermodilution (CO_TPTD_) in anesthetized piglets under normal conditions, volume resuscitation, inotrope infusion, and severe hemorrhage [28], the variations in the CO_TPTD_ greater than 15% lead to a change in the CO_EC_ in the same direction 93% of the time. The BA analysis shows a mean difference between the two techniques of −0.63 L/min with a SD of 0.64 L/min. The lower and upper LOA are −1.88 and 0.62 L/min with a percentage error of ±82.8%. The CO_EC_ measurements underestimate the high and overestimate the low CO_TPTD_ measurements. The large area of fat and muscle deposition around the cervical area of the piglets is presumed to have interfered with the EC’s functioning [28]. Another study evaluates the cardiac index (CI) measurements for the EC against PATD in anesthetized dogs undergoing experimental open-chest cardiovascular surgery for isolated right ventricular failure [24]. The overall bias and precision for CI_EC_ versus CI_PATD_ is −0.22 ± 0.52 L/min/m^2^ with LOA of from −1.25 to 0.81 L/min/m^2^. The difference between the methods is most pronounced for the low CI measurements compared with from normal to high CIs. The trend analysis for the CI_EC_ compared with the CI_PATD_ revealed a concordance of 88% with a significant correlation. The percentage error during the wide range of the CI_PATD_ recordings is from 19.4% to 41.2%, thus exceeding the acceptable value (<30%) [24,38]. The higher percentage error for the CO_EC_ in piglets and dogs is attributed to the species differences in the aortic arch’s location in the thorax, the skin resistance, and the thoracic cavity’s width [24,28]. Moreover, the angular bias slightly exceeds ±5° and a radial LOA over ±30°, thus suggesting poor trending ability for the EC. Importantly, the Electrical Velocimetry™ model uses the ‘volume of electrically participating tissue’ (V_EPT_) by using anthropometric measures such as the body mass and height to estimate the thorax’s electrically participating volume. Predominantly determined by the patient weight, the mass-based volumetric equivalent of the thoracic blood volume is determined using human subjects in stable normal states and unstable cardiopulmonary disease states [29,30]. Hence, we cannot eliminate the possibility that translating this patient constant based on human data to canines may have been a potential reason for the errors reported with the EC in the present study.

Thoracic impedance cardiography (ICG) is an easy noninvasive method for continuously measuring the CO. It delivers a low-amplitude, high-frequency electrical current across the thorax and the voltage is obtained by the electrodes applied to the skin of neck and thoracic wall. Assessing the CO is performed by detecting variations in the electrical resistance of the thorax during volumetric changes in the aorta over a cardiac cycle [8,9]. Several studies comparing the ICG and PATD have reported disagreements and ICG’s inaccuracy [48]. This traditional model of analyzing the bioimpedance signal was challenged, and a new model called Electrical Velocimetry^TM^ was developed that estimates the maximum rate of change in the thoracic electrical bioimpedance as the ohmic equivalent of the mean aortic acceleration [29,30]. The modified algorithm focuses on the aortic blood’s impedance and conductivity during a cardiac cycle, and neglects minor influences such as lungs, gas, and surrounding tissues. This improves EC’s reliability over the ICG, where the later method considers the volume of the surrounding tissues’ (e.g., thoracic fluid, tissue fluid volume, and pulmonary and venous blood) contributions in its calculations [29,30]. The major advantage of EC is its noninvasiveness, which causes it to be potentially useful in awake small animals. It also eliminates the recurrent need for calibration that minimally invasive lithium dilution and pulse contour analysis demand [8,9]. Performing it is very convenient and clinicians can be trained with ease. In this study, the time required by the researcher to place the ICON monitor on the dog and connect it to the patient data interface was 5–8 min. The ICON monitor is portable, user friendly, and the data acquisition and storage are simple and straightforward. EC’s limitations may be seen in similar scenarios as ICG, such as interference with surgical electrocautery, extreme movement, arrhythmias, magnetic resonance imaging units, and noise from mechanical ventilation. The presence of ascites may cause overestimation of the patient’s body weight, leading to inaccurate SV_EC_ and CO_EC_ measurements. Surgical manipulation of the upper abdomen may lead to shifts in the thoracic bioimpedance and affect the CO_EC_ values [8,9]. In veterinary medicine, the size of the animal may restrict the use of EC due to the increased distance between the neck and the thorax and the wider thoracic area in large animals. Additionally, while using the cardiotronic electrodes and EC monitoring for longer durations, the electrode surfaces may lose adhesion with the skin surface. This was observed in our study, so, to ensure constant electrode–skin surface contact, we used an adhesive, elastic bandage wrap around the electrodes to hold them in place and yield reliable CO readings.

Monitoring the fluid balance is a crucial concept in anesthesia and critical care units. In the past two decades, extensive work has been performed in the area of ‘fluid responsiveness’ to prevent fluid overload in patients. This can be favorable for decreasing morbidity and mortality, improving patient outcomes, and shortening hospitalization days. The EC-derived hemodynamic variables provide vital information regarding the fluid status and myocardial contractility. Thoracic intravascular and extravascular fluid content affect TFC values that are calculated from the thoracic electrical bioimpedance. Variations in TFC are known to reflect the total fluid changes [49,50]. In our study dogs, we find the TFC values closely follows the changes in the CO_PATD_ such that they decrease with hemorrhage and normalize to baseline values after autologous blood transfusion. In a hypervolemic state, after administration of a colloid bolus, the highest TFC values are reported. These findings are in accordance with the results from human subjects undergoing autologous blood harvest [49]. Both the initial and peak TFCs are shown to predict morbidity in critically ill children with respiratory failure and/or shock [50]. The FTC defines the systolic portion of the cardiac cycle, and its calculation is based on measuring the time between the aortic valve opening and closing during cardiac contraction. In anesthetized dogs, this measure can correlate with intravascular volume [33]. Similarly, we observe that FTC showed changes in the same direction as the CO_PATD_, indicating that this variable can correspond to acute changes in the hemodynamics. Mechanical ventilation induces cardiopulmonary interactions that can be beneficial for assessing the relationship between SV and the dynamic variables such as SVV and ΔPEP. During positive pressure ventilation, the greatest variations in SV happen in the face of hypovolemia. The EC-derived SVV can successfully guide fluid therapy in anesthetized dogs ventilated with different ventilation modes [26] as well as the ones undergoing emergency abdominal surgery and those diagnosed with pulmonary hypertension secondary to mitral valve disease [27]. We see the identical behavior of SVV in our ventilated dogs, where the variation in the SV increases in magnitude during absolute hypovolemia and significantly reduces during the blood and colloid administration. Similar to the findings from human studies [31,32], just like SVV, ΔPEP also acts as a surrogate to the CO_PATD_ in our study dogs and accurately tracks the hemodynamics. However, because the physiology behind the SVV and ΔPEP is analogous with the other dynamic variables, they may be unreliable during spontaneous ventilation, positive end-expiratory pressure, tidal volume <8 mL/kg, low respiratory system compliance, open-chest conditions, cardiac rhythm irregularities, right-sided heart failure, altered vascular tone, and intra-abdominal hypertension.

The PEP is the time interval from the onset of electrical stimulation of the ventricles (electrical systole) to the aortic valve opening. The LVET interval is the time from the aortic valve opening to closing (mechanical systole), denoting the aortic flow’s duration. Both these parameters represent a fine balance between the intrinsic contractile function, left ventricular preload, and afterload, but the PEP is also influenced by electrical activation [51,52,53]. The STR is a ratio of the electrical and mechanical systoles and is calculated as PEP/LVET. When ventricular systolic function is impaired and the ejection fraction is lowered, the time to generate sufficient pressure that can open the aortic valve increases while the ejection period reduces, thus resulting in higher STR values [51,52,53]. In this study, the ICON^TM^ and VIC values are significantly decreased during hypovolemia in the anesthetized dogs as expected due to the diminished ventricular preload. It is also possible that the systolic function may also have been disrupted due to the ongoing negative effects on the left ventricular–arterial coupling and mechanical efficiency as previously reported in anesthetized dogs [1,2]. When the blood volume is depleted, we observed an increase in the STR and PEP values and a decrease in the LVET readings. With blood transfusion and a colloid bolus, these changes are reversed, and the values normalize toward baseline. These results are consistent with a few human studies [51,52,53]. The PEP and LVET provide time-based assessments of the ventricular performance and to some degree depend on the heart rate or width of the QRS complex. Hence, the absolute values of PEP and LVET may require correction when the HR is altered. To dilute the HR’s influence, the STR values may be analyzed instead [54].

This study has the following limitations. The sample size was small, which causes the receiver operating characteristic curves to not generate. Hence, the predictive values, cut-offs, sensitivity, and specificity for the EC-derived variables are not reported. Considering this study primarily focuses on hemodynamics, we attempted to control factors such as the body temperature, use of drugs with cardiovascular effects, sympathetic stimulation, and anesthetic depth to isolate the impact of blood volume changes on the hemodynamic variables. Moreover, this work is an experimental study in healthy Beagle dogs. We realize this does not align with clinical scenarios and, hence, evaluating the EC in routinely anesthetized canine patients with systemic diseases is warranted. A larger study population and data set are required to confirm our study’s observations. The conveyance of the patient constant V_EPT_ derived from the human population to other species also needs further exploration. The sequence of subjecting the dogs to drastic variations in the blood volume was not randomized because this specific order was crucial to identify whether the EC can reflect the hemodynamic picture in these dogs. This was also essential to avoid the crossover effect of hypervolemia if induced prior to hypovolemia. The order of the PATD and EC was also not randomized during the data collection. Since the PATD measurements involved multiple 0.9% saline injections, we wanted to rule out any impact of the administered volume on the EC. Therefore, the EC recordings always preceded the PATD data acquisition.

## 5. Conclusions

This study is one of the first exploratory studies to report the assessment of EC-monitor-derived CO (i.e., ICON) and other hemodynamic variables during the induction and treatment of acute hemorrhagic shock in isoflurane-anesthetized dogs. A positive mean relative bias (27.7%) and slope (<1) about Y = X indicate that the EC underpredicts the CO when compared to the PATD. The agreement between the two methods significantly improves during acute hemorrhage, thus indicating that EC may be able to identify sudden variations in the hemodynamics during absolute hypovolemia in canine patients undergoing general anesthesia. Even though the concordance between the two methods is 0.65 and the percentage error observed during comparisons of the absolute measurements between PATD and EC is 49.4%, the trending ability of the CO_EC_ is consistently good throughout the experiment as compared to the CO_PATD_. The TFC, FTC, SVV, and ΔPEP can assess the volume status and act as surrogates to CO_PATD_, and this may have a clinical benefit in canine patients for monitoring fluid therapy. The ICON^TM^, VIC, PEP, and STR also provide critical hemodynamic information regarding the impact of acute hemorrhage on the contractile function. Electrical cardiometry is a pivotal noninvasive technique that derives hemodynamic variables that can guide anesthetists to anticipate, diagnose, and treat cardiovascular complications perioperatively, and may help improve the quality of patient monitoring and management in anesthetized dogs. Future studies demonstrating EC’s utility for canine patients undergoing cardiothoracic surgeries, sepsis, pulmonary edema, cardiogenic shock, cardiothoracic trauma, cardiopulmonary resuscitation, volume resuscitation, and receiving inotropic and vasopressor therapy will be fundamental in determining the clinical benefit of this noninvasive CO method in small animal medicine.

## Figures and Tables

**Table 1:** Mean *±* SD for cardiac output (CO) measurements using electrical cardiometry (CO_EC_) and pulmonary artery thermodilution (CO_PATD_) recorded in six healthy, isoflurane-anesthetized dogs during four hemodynamic events: (1) baseline; (2) withdrawal of 30 mL/kg blood volume inducing hemorrhage; (3) autologous blood transfusion; and (4) 20 mL/kg 6% hydroxyethyl starch 130/0.4 in 0.9% sodium chloride solution. Since the values for CO_PATD_ and CO_EC_ for the five timepoints under each event are not statistically significant, the mean ± SD values are calculated from the timepoint measurements to represent each event.

Hemodynamic Event	CO_PATD_ (L/min)	CO_EC_ (L/min)
Baseline	1.85 ± 0.3	1.32 ± 0.1
Hemorrhage	1.10 ± 0.1 *	1.05 ± 0.1 *
Autologous blood transfusion	2.19 ± 0.1 *^,†^	1.53 ± 0.1 *^,†^
6% hydroxyethyl starch infusion	3.09 ± 0.1 *^,†,‡^	2.16 ± 0.4 *^,†,‡^

* Significant difference (*p* < 0.05) from baseline, ^†^ significant difference (*p* < 0.05) from hemorrhage, and ^‡^ significant difference (*p* < 0.05) from autologous blood transfusion.

**Table 2:** Mean *±* SD for heart rate, stroke volume, thoracic fluid content, flow time corrected, stroke volume variation, and variation in pre-ejection period measurements recorded using electrical cardiometry (HR_EC_, SV_EC_, TFC, FTC, SVV, and ΔPEP) in six healthy, isoflurane-anesthetized Beagle dogs during four hemodynamic events: (1) baseline; (2) withdrawal of 30 mL/kg blood volume inducing hemorrhage; (3) autologous blood transfusion; and (4) 20 mL/kg 6% hydroxyethyl starch 130/0.4 in 0.9% sodium chloride solution. Since the values for HR_EC_, SV_EC_, TFC, FTC, SVV, and ΔPEP for the five timepoints under each event are not statistically significant, the mean ± SD values are calculated from the timepoint measurements to represent each event.

Hemodynamic Event	HR_EC_ (Beats/min)	SV_EC_ (mL)	TFC	FTC (ms)	SVV (%)	ΔPEP (%)
Baseline	90 ± 8	14.6 ± 1.5	18 ± 2	311 ± 13	9 ± 1	8.3 ± 1.0
Hemorrhage	143 ± 5 *	7.3 ± 0.8 *	13 ± 2 *	273 ± 11 *	23 ± 2 *	14.3 ± 1.1 *
Autologous blood transfusion	119 ± 3 *^,†^	12.8 ± 0.7 *^,†^	18 ± 2 ^†^	330 ± 8 *^,†^	9 ± 1 ^†^	7.7 ± 0.8 *^,†^
6% hydroxyethyl starch infusion	127 ± 3 *^,†,‡^	17.2 ± 3.0 *^,†,‡^	23.± 1 *^,†,‡^	360 ± 8 *^,†,‡^	5 ± 1 *^,†,‡^	4.3 ± 0.8 *^,†,‡^

* Significant difference (*p* < 0.05) from baseline, ^†^ Significant difference (*p* < 0.05) from hemorrhage, and ^‡^ significant difference (*p* < 0.05) from autologous blood transfusion.

**Table 3:** Mean *±* SD for contractility index (ICON™), variation in contractility (VIC™), systolic time ratio (STR), pre-ejection period (PEP), and left ventricular ejection time (LVET) measurements recorded using electrical cardiometry in six healthy, isoflurane-anesthetized Beagle dogs during four hemodynamic events: (1) baseline; (2) withdrawal of 30 mL/kg blood volume inducing hemorrhage; (3) autologous blood transfusion; and (4) 20 mL/kg 6% hydroxyethyl starch 130/0.4 in 0.9% sodium chloride solution. Since the values for ICON™, VIC™, STR, PEP, and LVET for the five timepoints under each event are not statistically significant, the mean ± SD values are calculated from the timepoint measurements to represent each event.

Hemodynamic Event	ICON^TM^	VIC^TM^ (%)	STR	PEP (ms)	LVET (ms)
Baseline	94 ± 13	19 ± 2	0.42 ± 0.10	109 ± 14	256 ± 14
Hemorrhage	67 ± 9 *	14 ± 2 *	0.58 ± 0.10 *	118 ± 6 *	203 ± 7 *
Autologous blood transfusion	104 ± 12 *^,†^	20 ± 1 ^†^	0.45 ± 0.1 ^†^	112 ± 11 ^†^	248 ± 12 ^†^
6% hydroxyethyl starch infusion	107 ± 10 *^,†^	19 ± 3 ^†^	0.41 ± 0.1 ^†^	108 ± 10 ^†^	259 ± 12 ^†^

* Significant difference (*p* < 0.05) from baseline, and ^†^ significant difference (*p* < 0.05) from hemorrhage.

## Data Availability

The data supporting the central findings of this research study are contained within the article. Other data pertaining to studying animals may be available on request and are subjected to evaluation on a case-by-case basis respecting the Virginia Polytechnic Institute and State University regulations and policies on data handling.
